# Overexpression of *mfp*A Gene Increases Ciprofloxacin Resistance in *Mycobacterium smegmatis*

**DOI:** 10.1155/2021/6689186

**Published:** 2021-03-22

**Authors:** Aura Falco, Carlos Aranaga, Ivan Ocampo, Howard Takiff

**Affiliations:** ^1^Grupo de Investigación en Microbiología, Industria y Ambiente (GIMIA), Facultad de Ciencias Básicas, Universidad Santiago de Cali, Cali, Colombia; ^2^Laboratorio de Genética Molecular, Centro de Microbiología y Biología Celular, Instituto Venezolano de Investigaciones Científicas, Km. 11, Carretera Panamericana, Caracas, Venezuela; ^3^Grupo de Investigación en Química y Biotecnología (QUIBIO), Facultad de Ciencias Básicas, Universidad Santiago de Cali, Cali, Colombia; ^4^Integrated Mycobacterial Pathogenomics, Institut Pasteur, Paris, France; ^5^Department of Tuberculosis Control and Prevention, Shenzhen Nanshan Centre for Chronic Disease Control, Shenzhen, China

## Abstract

Fluoroquinolones (FQs) are antibiotics useful in the treatment of drug-resistant tuberculosis, but FQ-resistant mutants can be selected rapidly. Although mutations in the DNA gyrase are the principal cause of this resistance, pentapeptide proteins have been found to confer low-level FQ resistance in Gram-negative bacteria. MfpA is a pentapeptide repeat protein conserved in mycobacterial chromosomes, where it is adjacent to a group of four highly conserved genes termed a *conservon*. We wished to characterize the transcriptional regulation of the *mfp*A gene and relate its expression to ciprofloxacin resistance in *M. smegmatis*. Reverse transcription PCR showed that *mfp*A gene is part of an operon containing the *conservon* genes. Using a transcriptional fusion, we showed that a promoter was located 5′ to the *mfp*EA operon. We determined the promoter activity under different growth conditions and found that the expression of the operon increases slightly in late growth phases in basic pH and in subinhibitory concentrations of ciprofloxacin. Finally, by cloning the *mfp*A gene in an inducible vector, we showed that induced expression of *mfp*A increases the ciprofloxacin Minimal Inhibitory Concentration. These results confirm that increased expression of the *mfp*A gene, which is part of the *mfp*EA operon, increases ciprofloxacin resistance in *M. smegmatis*.

## 1. Introduction

Fluoroquinolones (FQs) are the most important antibiotics in drug regimens used for treating Multidrug-Resistant Tuberculosis (MDR-TB) [1]. They exert a powerful bactericidal activity and can penetrate macrophages, where the tuberculosis-causing *Mycobacterium tuberculosis* bacilli reside [2]. There are, however, reservations regarding FQ use because resistant mutants can be selected in a remarkably short time [1]. The targets of the FQs are the DNA gyrase and DNA Topoisomerase IV. Mycobacteria only contain the gyrase, which catalyses negative supercoiling of bacterial DNA [2], and most FQ resistance is caused by substitutions in a few critical amino acids located in the Quinolone Resistance-Determining Regions (QRDR) of the GyrA and GyrB subunits, although efflux pumps have also been implicated in the development of resistance [3–5].

Another novel mechanism of FQ resistance involves proteins belonging to the Pentapeptide Repeat Family (PRF). In Gram-negative bacteria, the genes of the *qnr* family encode pentapeptide proteins that are generally found on transmissible plasmids and confer low-level FQ resistance [6–9]. The proteins of the pentapeptide family are composed almost entirely of a repeating five amino acid motif, in which every fifth amino acid is either leucine or phenylalanine [10]. Pentapeptides were initially associated with FQ resistance when it was found that a plasmid containing the *mfp*A (Mycobacterial Fluoroquinolone Resistance Protein) gene of *Mycobacterium smegmatis* increased the MICs to ciprofloxacin and sparfloxacin in this bacterium. This gene encodes a protein of 192 amino acids with 32 pentapeptide repeats in tandem [11]. In *M. tuberculosis*, the gene *Rv*3361c encodes a pentapeptide protein (termed MtMfpA) of 183 amino acids that has 67% amino acid identity with the *M. smegmatis* MfpA. The structure of the MtMfpA protein is a right-handed helix with the size, shape, and charge distribution reminiscent of B-form DNA, and it has been suggested that MfpA may compete with DNA for binding to the DNA gyrase. Because the FQs only bind to the gyrase when it is complexed with DNA, the binding of MfpA to the gyrase could either prevent the formation of the gyrase DNA complex, or replace DNA in a FQ-inhibited complex, thereby releasing the gyrase from FQ inhibition, and thus conferring resistance [12].

Very conserved *mfp*A genes are found on the chromosomes of all known mycobacterial genomes [10]. Their biological function is unknown, but they are accompanied at their 5′-end by a group of four highly conserved genes, termed a *conservon*, which have also been found in several other Actinobacteria, the class of bacteria to which mycobacteria belong. *Conservons* appear to be regulatory units that respond to unknown signals, and the fifth gene in the unit, where *mfp*A is found, is different in other Actinomycetes [13].

Because the FQs are an important drug for curing patients with MDR-TB [14, 15], we attempted to clarify the role of pentapeptide proteins in FQ resistance through genetic characterization of the *mfp*A gene in *M. smegmatis*. We found that the *mfp*A gene is part of a transcriptional unit with the four upstream *conservon* genes. Moreover, we showed that the promoter region for this operon, termed *mfp*EA, was at 5′-end of the *mfp*E gene. Finally, the *mfp*A gene was cloned into an inducible vector to increase its expression and thereby confirm that increased expression leads to increased FQ resistance. The results suggest a possible link between MfpA and FQ resistance in *M*. *smegmatis*, which could have important implications for FQ resistance in *M*. *tuberculosis*.

## 2. Materials and Methods

### 2.1. Bacterial Strains and Plasmids

*Escherichiacoli* XL1-Blue, used for cloning, was grown at 37°C in Luria–Bertani (LB) broth or on LB agar (Difco™, Cat. No. 244520, USA). *Mycobacteriumsmegmatis* strains were grown at 37°C in Middlebrook 7H9 broth (Difco™, Cat. No. 271310, USA) or 7H10 agar (Difco™, Cat. No. 262710, USA) supplemented with Tween (0.05%), glycerol (0.2%), and OAD (10%). The plasmids and bacterial strains used in this study are described in Table 1.

### 2.2. DNA Manipulation

The genomic DNA of *M*. *smegmatis* was isolated as described by van Soolingen et al. [19]. *Escherichia coli* was electroporated according to Hanahan et al. [20], while *M*. *smegmatis* was electroporated following the protocol of Jacobs et al. [21].

### 2.3. Cloning

**Figure 1:**
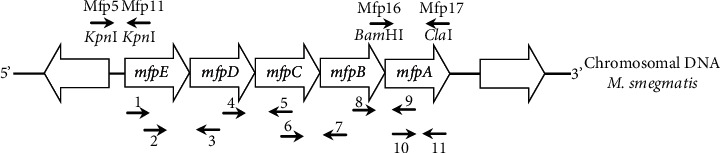
Schematic representation of the *mfp*EA operon. The arrows indicate the regions to be amplified with different primers.

A transcriptional fusion between the putative promoter region of the *mfp*EA operon and the *lac*Z gene in pJEM15 was constructed by PCR amplifying a 326 bp fragment at the 5′-end of the *mfp*E gene with primers mfp5 and mfp11 (Figure 1, Tables 1 and 2) and inserting it into the *Kpn*I restriction site on plasmid pJEM15. The *mfp*A gene was inserted into the expression vector pALACE by amplifying with primers mfp16 and mfp17 (Figure 1, Tables 1 and 2) and digesting with *Bam*HI and *Cla*I. The resulting constructs were verified by sequencing (Macrogen, South Korea) using the corresponding primers (Table 2).

### 2.4. Determination of Promoter Activity

The promoter activity of the transcriptional fusion was evaluated by the detection of beta-galactosidase activity in 7H9 media with Tween (0.05%), glycerol (0.2%), and OAD (10%). *Mycobacterium smegmatis* mc^2^155 was electroporated with plasmids pJEM15-5.11 and vector pJEM15 (Table 1). The promoter activity was determined by detecting Relative Fluorescence Units (RFU) produced by hydrolysis of the C2FDG (5-acetylamino di-beta-D-galactopyranoside) substrate (Molecular Probes, OR, USA). The assay was initiated by inoculating each strain into 5 ml of 7H9 supplemented with kanamycin (25 *μ*g/ml) and incubating at 37°C with agitation for 3 days. Subsequently, 0.1 ml of these cultures was inoculated into 10 ml of 7H9 with kanamycin (25 *μ*g/ml) and incubated at 37°C with agitation until reaching OD_600 nm_ 0.5. Fluorescence assays were performed in 96-well microtiter plates using 90 *μ*l (∼0.5 × 104 cells/well) of each culture (previously diluted 1:10^4^) mixed with 10 *μ*l of the C2FDG fluorophore (33 *μ*M). Bacteria were incubated with this substrate at 37°C for 96 hours, during which *β*-galactosidase activity was detected by exciting at a wavelength of 485 ± 20 nm and measuring emission at 530 ± 25, using a SpectraMax® Gemini XS instrument (Molecular Devices, CA, USA) [22]. In parallel, mycobacterial growth was followed measuring OD_600 nm_ as a function of time. These assays were done in triplicate, and the results show the standard deviation for each measurement.

### 2.5. Evaluation of Promoter Activity in Different Growth Conditions

The promoter activity of *mfp*EA operon under different conditions was evaluated as described above, with some modifications. *Mycobacterium smegmatis* mc^2^155 was transformed with plasmid pJEM15-5.11 and vector pJEM15 (Table 1) and grown for 96 hours in 7H9 in different conditions: 7H9 supplemented with ciprofloxacin (0.125 *μ*g/ml) and 7H9 adjusted to pH 8.0 by the addition of concentrated NaOH and then filter sterilized through a disposable polyethersulfone filter with a 0.45 mm pore size [23]. These assays were done in triplicate, and the results show the standard deviation for each measurement.

### 2.6. Inducible Expression of MfpA

MfpA expression was induced according to Lakshminarayan et al. [18]. In brief, the *M*. *smegmatis* strains used in this assay (mc^2^155 and mc^2^155*gyr*A, see Table 1) were electroporated with pALACE or pALACE-*mfp*A (Table 2) and grown overnight in 7H9 supplemented with 1% glucose. They were then washed once in 7H9 broth without glucose and resuspended to an OD_600nm_ 1.0 in 7H9 medium lacking glucose. Induction was initiated by adding acetamide to a final concentration of 0.02% and incubating overnight with constant agitation. Controls without acetamide were set up in parallel [18]. Subsequently, 20 *μ*l of 10^−4^ and 10^−5^ dilutions were plated on 7H10 supplemented with hygromycin (50 *μ*g/ml), acetamide (0.02%), and increasing concentrations of ciprofloxacin (0, 0.25, 0.5, 1, 2, 4, 8, 16, and 32 y 64 *μ*g/ml). These assays were done in triplicate. We used as positive and negative controls the antibiotics hygromycin (50 *μ*g/ml) and kanamycin (25 *μ*g/ml), respectively.

### 2.7. RNA Extraction and Reverse Transcription PCR

Total RNA from *M*. *smegmatis* was isolated as previously described [24], from bacteria grown to OD_600nm_ 0.5. Residual DNA in the mycobacterial total RNA preparations was removed using DNase I according to the manufacturer's instructions (Sigma-Aldrich, Inc., Darmstadt, Germany). A control with no reverse transcriptase (NRT) was used for a PCR reaction with primers to 16S rRNA [25] (Table 2) to confirm complete DNA removal. The M-MuLV Reverse Transcriptase (New England Biolabs, MA, USA) and random primers 9 (New England Biolabs, MA, USA) (Table 2) were used to produce cDNA according to the manufacturer's instructions. The cDNA was used to amplify the *mfp* genes, in triplicate, using ThermoPol® (New England Biolabs, MA, USA) and specific primers (see Figure 1 and Table 1: *mfp*ED, *mfp*DC, *mfp*CB, *mfp*BA, and *mfp*A). After PCR amplification, the fragments were sequenced with the amplification primers in the forward and reverse directions (Macrogen, Korea).

## 3. Results

### 3.1. Identification of the *mfp*EA Operon in *Mycobacterium smegmatis*

**Figure 2:**
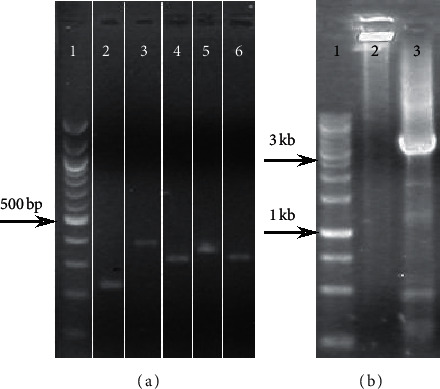
RT-PCR of the *mfp*EA operon. (a) Lane 1: MWM 100 bp ladder (NEB); Lane 2: RT-PCR using primers 10-11; Lane 3: RT-PCR using primers 8-9; Lane 4: RT-PCR using primers 6-7; Lane 5: RT-PCR using the primers 4-5; and Lane 6: RT-PCR using primers 2-3. (b) Lane 1: MWM O′ Gene Ruler 1 kb DNA ladder (Thermo Scientific); Lane 2: No Reverse Transcriptase control (NRT) using primers 1–11; and Lane 3: RT-PCR using primers 1–11.

We first used RT-PCR to determine whether *mfp*A is part of a transcriptional unit with the four upstream *conservon* genes (*mfp*E, *mfp*D, *mfp*C, and *mfp*B. See Figure 1 and Table 2). The amplification yielded a 221 bp fragment of the *mfp*A gene (Figure 2(a)), as well as fragments corresponding to the regions between *mfp*E and *mfp*A (4712 bp, see Figure 2(b)), *mfp*E and *mfp*D (312 bp), *mfp*D and *mfp*C (341 pb), *mfp*C and *mfp*B (311 bp), and *mfp*B and *mfp*A (375 bp) (Figure 2(a)), indicating that the five genes belong to the same transcriptional unit, which we term the *mfp*EA operon.

### 3.2. Promoter Activity for the *mfp*EA Operon

**Figure 3:**
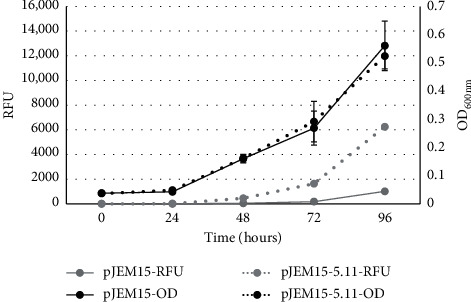
Promoter activity of the putative *mfp*EA operon. Curves labelled OD represent mycobacterial growth while the RFU curves indicate beta-galactosidase activity.

A 326 bp fragment including the 5′-end of the *mfp*E gene was cloned in front of the promoterless *lac*Z gene in vector pJEM15 to obtain the transcriptional fusion plasmid pJEM5-11 (Figure 1, Table 1). Subsequently, *M*. *smegmatis* mc^2^155 strains containing pJEM15 (control) or pJEM-5-11 (Table 1) were grown in 7H9 broth with C2FDG substrate (0.33 *μ*M), and promoter activity was measured as fluorescence resulting from the hydrolysis of C2FDG by beta-galactosidase. The fluorescence produced with plasmid pJEM-5-11 (grey dots) indicated that the cloned fragment contained a promoter whose activity correlated with bacterial growth (black dots) (Figure 3).

**Figure 4:**
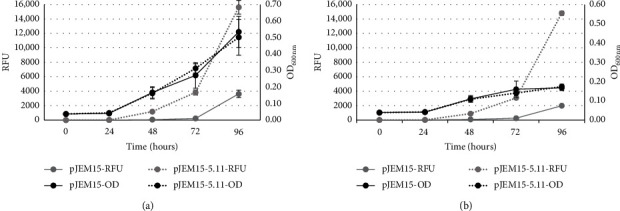
Promoter activity of the putative operon *mfp*EA operon in different growth conditions. (a) 7H9 (pH 8.0). (b) 7H9 with ciprofloxacin (0.125 *μ*g/ml). Curves labelled OD represent mycobacterial growth, while the RFU curves indicate beta-galactosidase activity.

The promoter activity was also evaluated in different growth conditions (see Materials and Methods). A slightly increased expression was seen in 7H9 growth media at pH 8.0 (Figure 4(a)) and with a subinhibitory concentration of ciprofloxacin (0.125 *μ*g/ml) (Figure 4(b)), but only after 96 hours of growth.

### 3.3. Determination of the Relation between *mfp*A and Ciprofloxacin Resistance

To determine the relation between *mfp*A and ciprofloxacin resistance, the *mfp*A gene was cloned into the pALACE vector to create plasmid pALACE-*mfp*A (Figure 1, Tables 1 and 2), in which the *mfp*A is expressed from an acetamide inducible promoter. The MICs for ciprofloxacin were compared between two strains of *M*. *smegmatis* containing either the empty vector or pALACE-*mfp*A: wild type (mc^2^155) and mc^2^155 *gyr*A D94G (Table 2). The induction of *mfp*A expression with acetamide produced increased ciprofloxacin resistance in both *M*. *smegmatis* strains. In the wild-type strain, the resistance to this antibiotic increased fourfold, while in the strain with the mutation in *gyr*A gene, it increased twofold (Table 3).

The relative changes in MIC^*∗*^ were expressed through the relationship (2)/(1).

## 4. Discussion

We have shown that the gene encoding the pentapeptide *mfp*A is the fifth gene in a transcriptional unit that also contains the four genes of the *conservon*, *mfp*EDCB. We have termed this the *mfp*EA operon. We also confirmed that increased transcription of *mfp*A increases FQ resistance, even in a strain containing a FQ resistance-conferring *gyr*A mutation. Although *conservons* are also found in several other actinobacteria, only in mycobacteria are they located 5′ to a pentapeptide encoding gene [10]. Judging by the domains identified in the four conservon encoded proteins, the unit appears to have a regulatory function: *mfp*B is similar to a signal recognition particle receptor beta subunit of a GTPase; *mfp*C contains a helix-turn helix motif; *mfp*D is similar to regulators with Ras-like GTPase activity; and *mfp*E is similar to signal transduction histidine kinases [13, 26, 27].

The fluoroquinolones act by inhibiting the activity of the gyrase, freezing the DNA gyrase complex with double-stranded breaks in the DNA that cannot be relegated. With the acetamide-induced promoter, we showed that increasing *mfp*A expression increases the FQ MICs, perhaps, as has been proposed, by mimicking and replacing the chromosomal DNA, thereby releasing the DNA trapped in the gyrase-FQ complex [10, 28, 29]. *mfp*A has been shown to interact with the DNA gyrase, an essential ATPase enzyme that introduces negative supercoils into the DNA molecule. Because mycobacteria contain no Topoisomerase IV, the gyrase presumably also performs its function of decatenating the two interlocking circular chromosomes resulting from DNA replication. Because it must perform these two functions, one of which is linked to DNA replication, it is possible that either the expression or the activity of MfpA and its interaction with the gyrase could be regulated by the *conservon* unit, but none of the conditions we tested appeared to substantially increase the transcription of the *mfp*EA operon. We reasoned that inhibition of the gyrase with ciprofloxacin might lead to increased transcription of the *mfp*EA operon to mitigate the effects of the drug, but we found just a slight increase in promoter activity only after 96 hours of culture. Other studies have shown that the expression of *qnr* genes increases with the SOS response [30], and the FQ's have been shown to induce the SOS response [31], but the increased promoter activity after 96 hours does not appear typical of an SOS response. We tentatively conclude that if the *conservon* somehow regulates *mfp*A activity, the regulation is not at the level of transcription. It is possible that the *mfp*EA operon has additional, internal promoters that are regulated, or the conservon acts at a posttranscriptional level by somehow modulating MfpA function through an alteration of GTPase or ATPase activity suggested by the conserved domains in the putative proteins encoded by the conservon genes *mfp*B and *mfp*D, respectively.

With the *lac*Z fusion, we showed that there was a promoter upstream of *mfp*E that expresses the whole operon, including *mfp*A, but promoter motifs are heterogeneous in mycobacteria and we could not identify the sequence of the putative promoter [32]. However, any mutation that increases promoter activity would raise *mfp*A expression and thereby increase FQ resistance. In *M. tuberculosis*, the arrangement of the *mfp*EDCBA genes is the same as in *M. smegmatis*, and *mfp*A has been shown to increase FQ resistance in *M*. *tuberculosis* family member *M*. *bovis* BCG [11]. *mfp*A could therefore contribute to intrinsic levels of resistance to the FQs in *M*. *tuberculosis* [3], and any mutation in the *mfp*EA promoter that increases its expression would be expected to increase FQ resistance. If these mutations occur in strains with FQ resistance mutations in the gyrase, they might increase the MIC in an additive manner. We found that induced expression of *mfp*A doubled the ciprofloxacin MIC in a strain containing the *gyr*A D94G mutation, a high-level resistance mutation frequently found in FQ resistant strains of *M. tuberculosis*. Alternatively, if a mutation augmenting the expression of *mfp*A occurs before QRDR mutations, it could increase the tolerance to low levels of FQs and thereby increase the frequency of high-level resistance gyrase mutations. To our knowledge, however, mutations in the *mfp*EA operon have not been described in drug-resistant *M*. *tuberculosis*, although it is not clear that this operon has been considered a site deserving careful examination.

## 5. Conclusions

The *mfp*A gene, encoding the pentapeptide MfpA, is the fifth gene in the *mfp*EA operon that contains the four genes of the *conservon*, *mfp*EDCB, and is expressed from a promoter located 5′ to *mfp*E. Overexpression of the *mfp*A gene increases ciprofloxacin resistance in *M*. *smegmatis*, even in strains with high-level FQ-resistance *gyr*A mutations.

## Figures and Tables

**Table 1:** Strains and plasmids used in this work.

Strains and plasmids	Relevant characteristics	References
*E*. *coli* XL1-Blue	*rec*A, *end*A, *gyr*A, *thi*-1, *hsd* R17, *sup*E44, *rel*A, *lac* [F′ *pro*AB *lac*I^q^ZΔM15 Tn10 (Tet^R^)].	Stratagene, CA, USA
*M*. *smegmatis* mc^2^155	Mutant, transformable strain of *M*. *smegmatis* derived from the strain mc^2^6.	Snapper et al. [16]
*M*. *smegmatis mc*^2^155 *gyr*A D94G	FQ-resistant mutant strain of *M. smegmatis* mc^2^155 with the *gyr*A D94G substitution.	Our laboratory
pJEM15	Cloning and shuttle vector (9.5 kbp) with a promoterless *lac*Z gene. Kan^R^ (25 *μ*g/ml).	Timm et al., [17]
pJEM15-5.11	pJEM15 cloning vector with the putative promoter region of the *mfp*EA operon.	This study
pALACE	Expression and shuttle vector (8221 bp) with a strong promoter (Pace) induced with acetamide. Hyg^R^ (50 *μ*g/ml).	Lakshminarayan et al. [18]
pALACE-*mfp*A	pALACE vector with the *mfp*A gene of *M*. *smegmatis*.	This study

**Table 2:** Primers used in this study.

Primer name	Primer sequence
mfp5^*∗*^	TTTTTTGGTACCTACCGGTATTCGGCGCGAT
mfp11^*∗*^	TTTTTTGGTACCCGCCCAACCGTGCCATCG
mfp16^*∗*^	TTTTTTGGATCCGCTGCCCGGCTGAGGCTT
mfp17^*∗*^	TTTTTTATCGATCTGCTCGCGGTGAGAAAC
Random primers 9	(d(N)_9_ where N = A, C, G, T)
1	TTGCAGCAGCGGGTGGATTC
10	GACGAGGATCTGGAGCCGGCAT
11	GAGCGTGAAGTCGCACTCGA
2	AAACGACAACGGTGCCGAGG
3	GCGTTCGACCGGCATGTTCT
4	GGCTACGTGCTGCAATCGGT
5	GCGCGTTGGGTGTGTCGAGA
6	GCGTGGCGAGAGTGCTGATC
7	GTGGTGGTGCGTTTGTCGGG
8	GCCAAAGCATCCCACGCACG
9	CGTGCTGTGCCAGATGGTGC
16S forward	CCAGCAGCCGCGGTAATACG
16S reverse	ATCGG(C/T)TACCTTGTTACGACTTC

^*∗*^Underlined letters indicate restriction sites. Figure 1 shows target sites of primers in *mfp*EA operon.

**Table 3:** Effect of *mfp*A overexpression on ciprofloxacin MIC.

Strains	MIC (*μ*g/ml) ciprofloxacin		Relative change in the MIC^*∗*^
	pALACE (1)	pALACE-*mfp*A (2)	
mc^2^155	0.25	1	4
mc^2^155 *gyr*A D94G	1	2	2

## Data Availability

The data used to support the findings of this study are included within the article and are available from the corresponding author upon request.
